# Acute dengue myositis with rhabdomyolysis and acute renal failure

**DOI:** 10.4103/0972-2327.70882

**Published:** 2010

**Authors:** Sourya Acharya, Samarth Shukla, S. N. Mahajan, S. K. Diwan

**Affiliations:** Department of Medicine, J.N Medical College, DMIMS University, Sawangi (Meghe), Wardha - 442 004, Maharashtra, India; 1 Department of Pathology, J.N Medical College, DMIMS University, Sawangi (Meghe), Wardha - 442 004, Maharashtra, India

**Keywords:** ARF, creatine kinase, flavivirus, encephalitis, myositis quadriparesis

## Abstract

Dengue is an acute mosquito-borne infection caused by dengue viruses from the genus flavivirus. Neurologic complications have been attributed chiefly to metabolic alterations and to focal and sometimes massive intracranial haemorrhages, but anecdotal cases and limited case series have indicated the possibility of viral CNS and skeletal muscle invasion causing encephalitis and myositis. We present a case of a 40-year-old male who presented with severe dengue myositis resulting in quadriparesis, respiratory failure and acute renal failure with red urine. His elevated serum creatine kinase (CK), serum and urine myoglobin levels justified rhabdomyolysis as the cause of acute renal failure. A muscle biopsy revealed inflammatory myositis. He required ventilator support for respiratory failure and was treated conservatively. This case highlights the severe and persistent muscle involvement in dengue which is a rarity.

## The Case

**Figure 1:**
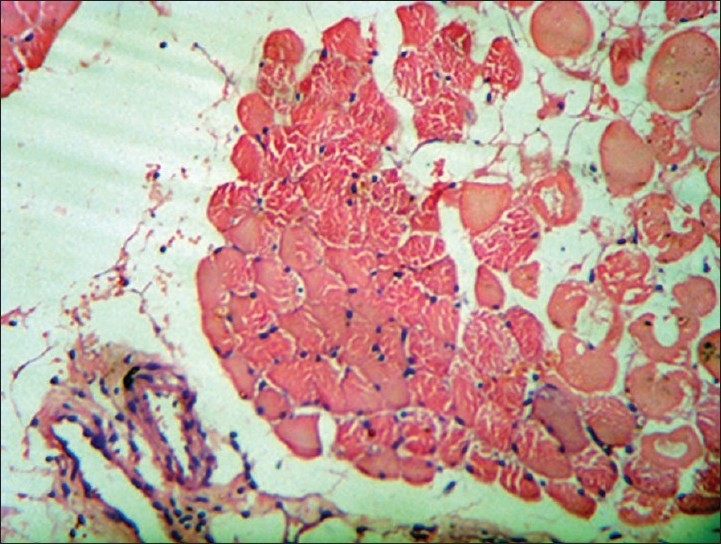
H and E stained slide from quadriceps muscle biopsy shows perifascicular infl ammatory infi ltrates with few areas showing necrosis. Suggestive of infl ammatory myopathy. Low magnification, ×10

A 40-year-old male, manual labourer presented with fever and myalgia of 4 days duration. He was febrile, conjunctival congestion and diffuse muscle tenderness were present, limb movements were painful but power was reasonably normal. Next day he developed acute flaccid, hyporeflexic, pure motor quadriparesis, which progressed to bilateral pharyngeal muscle weakness, head drop and respiratory insufficiency over next 12 h. Power in the proximal group of muscles was grade 2/5 and in distal muscles was 4/5. He developed dark coloured urine and oliguria. On investigations: CBC was normal, the absolute platelet count was 88,000/mm^3^ and urine Dipstik test was positive for blood with microscopy showing 2 red cells/HPF. Microscopic examination showed pigmented granular casts. Serum total CK was 29,000 (normal 40-210 U/L). Serum creatinine was 2.6 mg%, serum myoglobin 442 μg/L (normal 19-92 μg/L) and urine myoglobin 226 ng/L (normal < 35 ng/L). Dengue antibody titre Ig M ELISA was strongly positive suggesting recent infection. Leptospira antibody, Hb S Ag and HCV were negative. EMG showed polyphasic action potentials with short duration and latencies and increased interference pattern suggesting myopathic injury. USG KUB was done which revealed normal-sized kidneys with slightly increased cortical echos. A muscle biopsy revealed perifascicular myonecrosis [Figure 1].

## DISCUSSION

Dengue virus can affect the brain, spinal cord, spinal roots, peripheral nerves and muscles.[1–3] The neurological manifestations of dengue fever include mononeuropathies, polyneuropathies, encephalitis, Guillain-Barré syndrome and rhabdomyolysis.[4] In a large study from India[5] it was observed that, neurologic manifestations of dengue fever presented in two major categories, encephalopathy and pure motor quadriparesis. The pure motor quadriparesis group had normal NCS, myopathic EMG and raised serum CK suggesting myositis. All the patients in the myopathy group improved, but the prognosis of encephalopathy group was poor with two deaths. Renal failure was seen in one patient in the encephalopathy group, but none of the patients in the myopathy group had renal failure irrespective of clinical and biochemical evidence of myositis, suggesting that total CK levels may not predict renal failure in cases of dengue myositis. It is a biomarker of myositis with or without clinical evidence of muscle paralysis and can occasionally be elevated in patients with dengue encephalopathy without obvious myositis.[6] Persistent dengue myositis can occur even after serological recovery of the infection because of corticosteroids.[7] The probable cause of prolonged, persistent inflammation in our case could be the vigorous manual labour done by the patient for 2 days after developing fever and ongoing myalgia, which exacerbated the inflammation. One study emphasizes the importance of serum CK in patients with fever and myalgias with or without overt muscle weakness and concludes that increased serum CK levels in the context of fever and myalgias should be considered as dengue fever even before serological confirmation, with a positive predictive value of 84% and negative predictive value of 98%.[8] Above studies indicate the importance of serum CK as a diagnostic tool in dengue myositis, but it does not predict the severity of the muscle involvement and paralysis. Renal injury comprising increased creatinine, proteinuria, glomerulonephritis, acute kidney injury (AKI) and haemolytic uraemic syndrome has been reported in dengue patients.[9–13]

Myositis associated with viral infection is a well-described entity but few reports are with dengue virus infection.[14] Possible mechanisms are direct viral invasion of the muscle fibres and toxin generation. The more likely cause of myositis by a dengue virus is by the production of myotoxic cytokines, particularly tumour necrosis factor (TNF) released in response to viral infection. Dengue virus infection had been shown to increase production of TNF in humans.[15] Studies of muscle biopsies in patients with dengue reported varied findings from inflammatory infiltrate to foci of myonecrosis.[16] Elevated CK levels remain the most sensitive indicator of myositis. Though a rare complication of dengue, it should be kept in mind by physicians as the incidence of dengue infection increases.

## References

[1] Solomon T, Dung NM, Vaughn DW, Kneen R, Thao LT, Raengsa-kulrach B (2000). Neurological manifestations of dengue infection. Lancet.

[2] Miagostovich MP, Ramos RG, Nicol AF, Nogueira RM, Cuzzi-Maya T, Oliveira AV (1997). Retrospective study on dengue fatal cases. Clin Neuropathol.

[3] Warke RV, Becerra A, Zawadzka A, Schmidt DJ, Martin KJ, Giaya K (2008). Efficient dengue virus (DENV) infection of human muscle satellite cells upregulates type I interferon response genes and differentially modulates MHC I expression on bystander and DENV-infected cells. J Gen Virol.

[4] Davis JS, Bourke P (2004). Rhabdomyolysis associated with dengue virus infection. Clin Infect Dis.

[5] Mishra UK, Kalita J (2006). Spectrum of Neurological Manifestations of Dengue in India. Dengue Bull.

[6] Beauvais P, Quinet B, Richardet JM (1993). Dengue apoptosis of two cases. Arch Fr Pediatr.

[7] Finsterer J, Kongchan K (2006). Severe, persisting, steroid responsive Dengue myositis. J Clin Virol.

[8] Said SM, Elsaeed KM, Zakareya A (2008). Benign acute myositis in association with acute dengue viruses. Infections Egypt J Neurol Psychiat Neurosurg.

[9] Lima EQ, Gorayeb FS, Zanon JR, Nogueira ML, Ramalho HJ, Burdmann EA (2007). Dendue haemorrhagic Fever induced acute kidney injury without hypotension, hemolysis and rhabdomyolysis. Nephrol Dial Transplant.

[10] George R, Liam CK, Chua CT, Lam SK, Pang T, Geethan R (1988). Unusual clinical manifestations of dengue virus infection. Southeast Asian J Trop Med Public Health.

[11] Hommel D, Talarmin A, Reynes JM, Hulin A (1999). Acute renal failure associated with dengue fever in French Guiana. Nephron.

[12] Futrakul P, Poshyachinda V, Mitrakul C, Kun-Anake C, Boonpuc-knavig V, Boompucknavig S (1973). Renal involvement and reticulo-endothelial-system clearance in dengue hemorrhagic fever. J Med Assoc Thai.

[13] Karakus A, Banga N, Voorn GP, Meinders AJ (2007). Dengue shock syndrome and rhabdomyolysis. Neth J Med.

[14] Gunasekera HH, Adikaram AV, Herath CA, Samarasinghe HH (2000). Myoglobinuric acute renal failure following dengue viral infection. Ceylon Med J.

[15] Gagnon SJ, Mori M, Kurane I, Green S, Vaughn DW, Kalayanarooj S (2002). Cytokine gene expression and protein production in peripheral blood mononuclear cells of children with acute dengue virus infections. J Med Virol.

[16] Malheiros SM, Oliveira AS, Schmidt B, Lima JG, Gabbai AA (1993). Dengue. Muscle biopsy findings in 15 patients. Arq Neuropsiquiatr.

